# Volumetric variation of peri-implant soft tissues in convergent collar implants and crowns using the biologically oriented preparation technique (BOPT)

**DOI:** 10.4317/medoral.22946

**Published:** 2019-08-18

**Authors:** Guillermo Cabanes-Gumbau, Agustín Pascual-Moscardó, David Peñarrocha-Oltra, Berta García-Mira, Javier Aizcorbe-Vicente, María Peñarrocha-Diago

**Affiliations:** 1 MD, DDS, MS, PhD. Collaborating Professor of the Master of Oral Surgery and Implantology. Valencia University Medical and Dental School. Valencia, Spain; 2 DDS, MS, PhD. Assistant Professor of Dental Materials. Valencia University Medical and Dental School. Valencia, Spain; 3 DDS, MS, PhD. Collaborating Professor and Doctor of Oral Surgery. Valencia University Medical and Dental School. Valencia, Spain. Investigator of the Patología y Terapéutica Odontológica y Maxilofacial group of the Instituto de Investigación Biomédica de Bellvitge (IDIBELL). Barcelona, Spain; 4 DDS, MS, PhD. Collaborating Professor and Doctor of Oral Surgery. Valencia University Medical and Dental School. Valencia, Spain; 5 DDS. Resident of the Master of Oral Surgery and Implantology. Valencia University Medical and Dental School. Valencia, Spain; 6 DDS, MS, PhD. Assistant Professor of Oral Surgery. Valencia University Medical and Dental School. Valencia, Spain. Investigator of the Patología y Terapéutica Odontológica y Maxilofacial group of the Instituto de Investigación Biomédica de Bellvitge (IDIBELL). Barcelona, Spain

## Abstract

**Background:**

To evaluate the changes in the peri-implant soft tissues of convergent collar implants with biologically oriented preparation technique (BOPT) crowns, 10 months after loading.

**Material and Methods:**

A pilot study was carried out from January 2016 to October 2017 involving 14 patients with one or two implants in the posterior mandibular sector. A total of 32 convergent collar implants were placed using a non-submerged protocol. Three months later the provisional cemented crowns were fitted using the BOPT approach with the finish line 1-1.5 mm below the gingival margin, simulating coronal emergence of a natural tooth. The soft tissue changes were measured with an intraoral scanner at two different timepoints: a) on the day of provisionalization, before prosthetic loading; and b) 10 months later without the provisional prosthesis. The STL files were superimposed and the soft tissue changes were recorded using a color scale with measurement of the volumetric changes in mm3.

**Results:**

A mean increase in peri-implant mucosal volume of 64.7 mm3 was observed in 29 implants. The zones with the greatest increase in soft tissue volume were the papillae of implants with adjacent teeth and the peri-implant buccal margin. Three implants showed a mean decrease in soft tissue volume of -25.1 mm3.

**Conclusions:**

The fitting and design of crowns using the biologically oriented preparation technique (BOPT) over convergent collar implants affords a significant increase in peri-implant soft tissue volume both at the level of the papillae and in the buccal margin.

** Key words:**Dental implants, one-piece dental implants, convergent collar implants, soft tissue volume, peri-implant mucosa, BOPT, vertical preparation, shoulderless abutments, emergence profile, intraoral scanner, profilometric analysis.

## Introduction

Implant success in the aesthetic sector is determined not only by osseointegration but also by the stability of the peri-implant soft tissues, affording a natural appearance to the rehabilitation and preventing bone reabsorption (1). The presence of a healthy peri-implant mucosal interface has been associated with protection against marginal bone loss and long-term implant success (2). The quality of this mucosa is determined in part by the prosthetic accessory materials in contact with it and the topography of the implant (3,4). In fact, the response of these tissues has been the subject of debate in systematic reviews published to date (5-7). The development of new dental implants, prosthetic abutments and crowns offers novel surfaces and designs capable of improving soft tissue insertion, with a view to avoiding microbial contamination of vital bone (8,9).

Marginal bone loss around implants is related to different parameters such as the thickness of the peri-implant mucosa (10,11), the inter-implant distance (12), the macro- and microscopic characteristics of the implant (4), and the design of the implant-abutment interface (13).

The biologically oriented preparation technique (BOPT) in implantology aims to allow the clinician to decide and adapt the marginal level of the peri-implant soft tissues, modifying the emergence profile of the prosthetic crown (14,15). The BOPT concept has been described as affording an adaptive profile of the soft tissues, which invade the sulcus in a controlled manner (14). With this technique the collagen fiber distribution appears to increase mucosal fixation around the teeth (and implants) and increase soft tissue stability over the long term, with the aim of maintaining peri-implant bone protection. The use of prosthetic abutments over convergent collar implants results in a prosthesis without a finishing line or margin, in which the emergence profile of the crown shapes the gingival margin.

The BOPT concept referred to dental prosthesis has been transferred to cemented implant prosthesis. The convergent collar portion of the implant-abutment assembly, together with the BOPT design crowns, has been suggested to provide positive outcomes such as the prevention of bone remodeling and preservation of the alveolar ridge (16), adequate peri-implant tissue stability (15), and improved peri-implant function and aesthetics, without the need for more invasive and costly bone or soft tissue regeneration techniques (17).

Volumetric changes have been compared from three-dimensional (3D) surface scans of the impression models before the extractions and 5 years after implant placement, superimposing the STL files to quantitatively evaluate the changes in tissue contour and soft tissue recessions measured up to the most apical portion of the implant and mucosal margin. The alterations in volume have been digitally measured as mean distance (mm) or volume (mm3) increase or loss in animals (18) and in humans (19).

The aim of the present pilot study was to evaluate volumetric soft tissue changes after using implants with a convergent collar and with BOPT prosthetic components and crowns in posterior mandibular sectors 10 months after loading.

## Material and Methods

-Study design and patient selection

**Figure 1:**
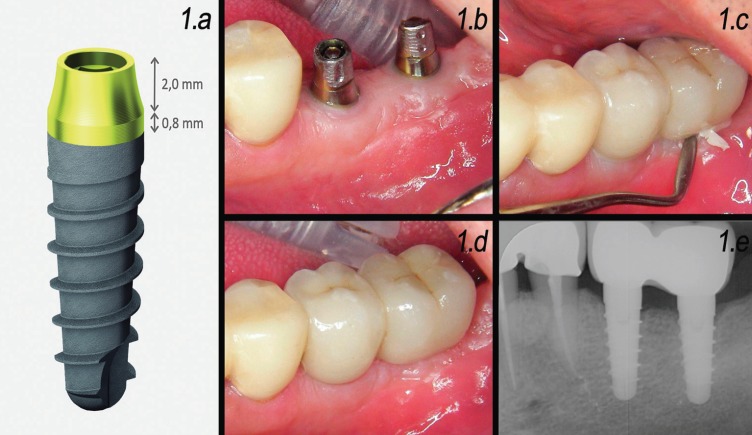
1.a: Schematic representation of the implant with a machined-surface convergent collar (Prama®, Sweden & Martina, Padua, Italy) used in the study. 1.b: Representative clinical case; peri-implant mucosa prior to loading. 1.c: Cementation of definitive crowns. 1.d: Intraoral aspect of peri-implant tissues 1 month after loading. 1.e: 1-month radiographic control.

A pilot study was carried out in the Oral Surgery Unit of the University of Valencia (Valencia, Spain) from January 2016 to October 2017. A total of 17 consecutive patients requiring single or partial restorations supported by up to two implants in the posterior mandibular sector were recruited. All the patients were treated with convergent collar implants and restored with BOPT cemented crowns (Fig. 1). The study was approved by the Ethics Committee of the University of Valencia (Ref. H1514988605552), and was carried out following the recommendations of the Declaration of Helsinki. All patients were informed about the study and gave written consent to participation in the trial. The inclusion and exclusion criteria are specified in  Table 1.

**Table 1:**
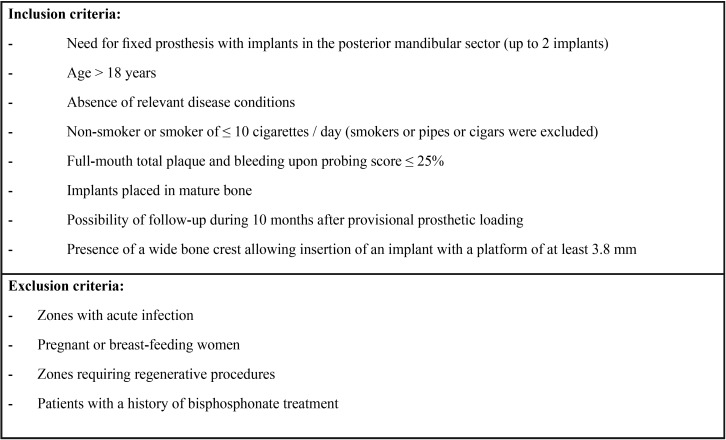
Study inclusion and exclusion criteria.

-Surgery and rehabilitation

All surgeries and prosthesis were performed by the same practitioner. The patients underwent professional dental hygiene in the clinic one week before implant surgery. The implants were placed using the same surgical protocol under local anesthesia with 4% articaine and adrenalin (1:100,000). A crestal incision was made in the adhered mucosa of the edentulous area, including the sulcus of the adjacent teeth, and a full-thickness mucoperiosteal flap was raised. Implants with a machined-surface convergent collar (Prama®, Sweden & Martina, Padua, Italy) were placed at supracrestal position. A non-submerged protocol was used, and the flap was replaced and sutured around the healing abutments. Preoperative antibiotic prophylaxis was provided one hour before surgery in the form of amoxicillin 2 g or clindamycin 600 mg in the case of patients allergic to the former drug. Postoperative medication included ibuprofen tablets (600 mg) in the event of pain, and 0.12% chlorhexidine digluconate rinses for one minute, twice a day during one week. The sutures were removed one week after surgery.

After a healing period of three months, the cemented acrylic provisional restorations were prepared using the BOPT approach. The margin of the restorations was 1-1.5 mm apical to the peri-implant soft tissue margin, simulating the coronal emergence of a natural tooth. The restorations were positioned on the implants with temporary cement (Premier Implant Cement, Premier®, Plymouth Meeting, PA, U.S.A.).

Data recording and follow-up

Age, gender, smoking habit and gingival biotype were collected for each patient. The position, diameter and length of each implant was documented. Implant success in the course of follow-up was evaluated based on the criteria of Buser (20).

**Figure 2:**
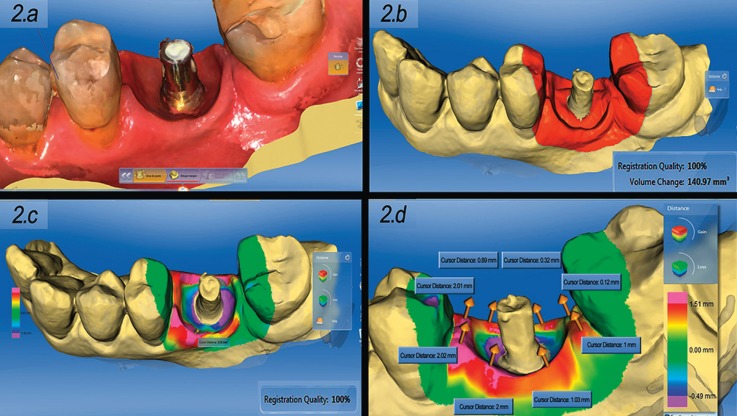
2.a: Intraoral scan of the gingival profile 10 months after crown placement. 2,b: Superimposition of images at T0 and T1 for quantitative volumetric evaluation (in mm3, using the Oracheck application) of the variations in gingival profile. 2.c: Superimposition of images at T0 and T1 for qualitative volumetric evaluation (based on a color scale using the Oracheck application) of the variations in gingival profile (blue = loss of volume, green = no change, red = increase in volume). 2.d: The values reflected in figures 4 and 5 are obtained from the average of this 8 measurement points.

An intraoral scanner (CEREC OMNICAM, Sirona®, Salzburg, Austria) was used to measure the soft tissue changes at two different timepoints: a) T0 (on the day of provisionalization, scanning was made before prosthetic loading); and b) T1 (10 months later, scanning was made without the provisional prosthesis) (Fig. 2.a). Using the OraChek® application (CEREC OMNICAM, Sirona®, Salzburg, Austria), the images corresponding to T0 and T1 were superimposed to evaluate the three-dimensional soft tissue changes during follow-up (Fig. 2.b). The study parameters were: a) distance gain coded as yellow, orange, red and pink, with pink being the zone of maximum dimensional increase. Reductions in distance in turn were coded as blue and violet, while a green color indicated no changes in soft tissue volume (Fig. 2.c); and b) quantification of the soft tissue volumetric changes in mm3. Also the quantitative change in coronal marginal height for the distal papilla, mesial papilla, buccal margin and lingual margin were obtained from the average of the 8 measurement points shown in Figure 2.d.

**Figure 3:**
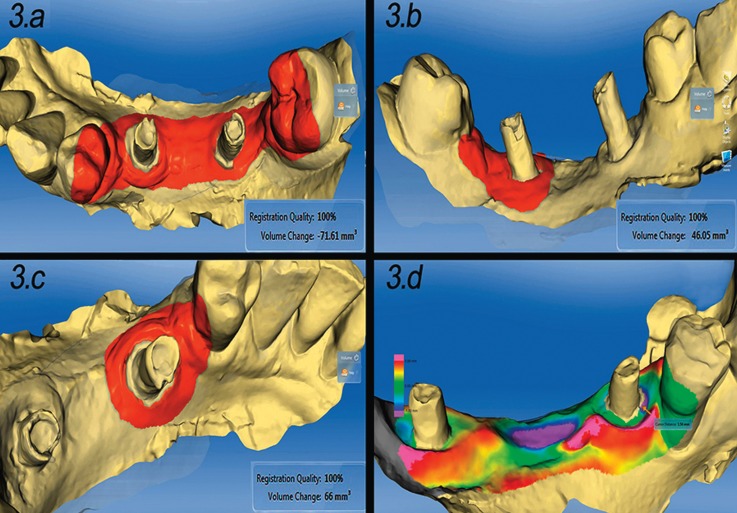
3.a: In implant-supported bridges, the inclusion of the pontic area in quantification of gingival variations yields negative volume values. 3.b: Only including distal peri-implant area (without the pontic) the volumetric quantitative variation between T0 and T1 was positive. 3.c: Only including mesial peri-implant area (without the pontic) the volumetric quantitative variation between T0 and T1 was positive. 3.d: Illustrative scans at T0 and T1 for qualitative volumetric evaluation of implant-supported bridges, in which, soft tissue volume loss is usually seen in the pontic area.

The following zones were delimited for the measurement of the soft tissue volumetric changes:

• Mesiodistal: between the closest cuspids of the adjacent teeth. In the cases of implants placed with no distal teeth, the distal boundary was fixed at 5 mm distal to the end of the crown of the implant.

• Occlusal-apical: a perpendicular line located 4 mm below the gingival margin of the crown.

• The volumetric changes (in mm3) were obtained individually for all the implants. In the cases of implant-supported bridges, the data corresponding to the entire bridge were also recorded in order to assess the volumetric changes caused by the pontic (Fig. 3.a-d).

All measurements were made by two independent examiners (G.C. and A.P.).

Statistical analysis

Descriptive analysis was used to calculate the main statistics: mean, standard deviation (SD), minimum and maximum and median (for continuous variables), and absolute and relative frequencies (for categorical variables).

Inferential analysis was used to determine whether the dimensional changes between T0 and T1 were relevant. The Kolmogorov-Smirnov test was applied to confirm normal data distribution. Nonparametric tests were used due to the limited sample size:

▪ One-sample t-test to determine possible dimensional changes between the two study timepoints, with calculation of the corresponding 95%CI.

▪ Estimation of the 95%CI of the median.

▪ Paired t-test to determine whether the mean change was similar at mesial and distal (or buccal and lingual) level.

▪ The Wilcoxon test to determine whether the distribution of the values corresponding to change were similar at the abovementioned levels.

▪ The Mann-Whitney U-test to contrast the equality of distributions in two independent groups (unit crowns and crowns conforming a bridge; thin and thick gingival biotype).

▪ The Kruskal-Wallis test to contrast the equality of distributions in more than two independent groups.

The level of statistical significance was defined as 5% (α=0.05). Statistical significance was represented by *p* < 0.05, while *p* ≥ 0.05 was taken to indicate a lack of statistical significance.

For a one-sample t-test with a confidence level of 95%, and considering an effect size d = 0.5 (mean), the statistical power was 79%. This effect size is equivalent to a difference of 0.25 mm in the measurement of the papilla between the two timepoints, assuming a standard deviation of 0.5 mm.

## Results

Out of a total of 17 initially selected patients, one was excluded because horizontal regenerative procedures were required and two patients were lost to follow-up. The final study sample thus consisted of 14 patients (10 females and 4 males) with a mean age of 60.4 years. Seven patients presented a normal or thick gingival biotype while 7 presented a thin biotype.

A total of 32 implants were placed. Twenty-four implants support single restoriations (in case of two adyacent implants single crowns were splinted) while 8 implants supported 3-unit fixed bridges with two crowns and a pontic crown. The implants were placed in the premolar or molar areas of the mandible. Twenty-seven implants had a diameter of 3.8 mm and 5 had a diameter of 4.25 mm.

The duration of follow-up from the first to the second scan was 10 months. At the follow-up visit, all the implants were clinically osseointegrated and stable, with no signs of inflammation. The resulting success rate was 100%.

Dimensional changes of the papilla

The mean mesial gain was 0.70 ± 0.55 mm (range 0-2 mm) (95%CI 0.50-0.90), indicating significant advancement (*p*<0.001; one-sample t-test). Of note is the fact that the median was only slightly greater: 50% of the implants showed advances of over 0.75 mm. The 95%CI of the median was 0.3-0.8, which likewise suggests significant gain.

The mean distal gain was 0.45 ± 0.50 mm (range 0-1.5 mm) (95%CI 0.27-0.63), indicating significant advancement (*p*<0.001; one-sample t-test). The median was only 0.20 mm, thus indicating that the gain was great for a few implants. The 95%CI of the median was 0.0-0.6, which does not indicate significant gain.

**Figure 4:**
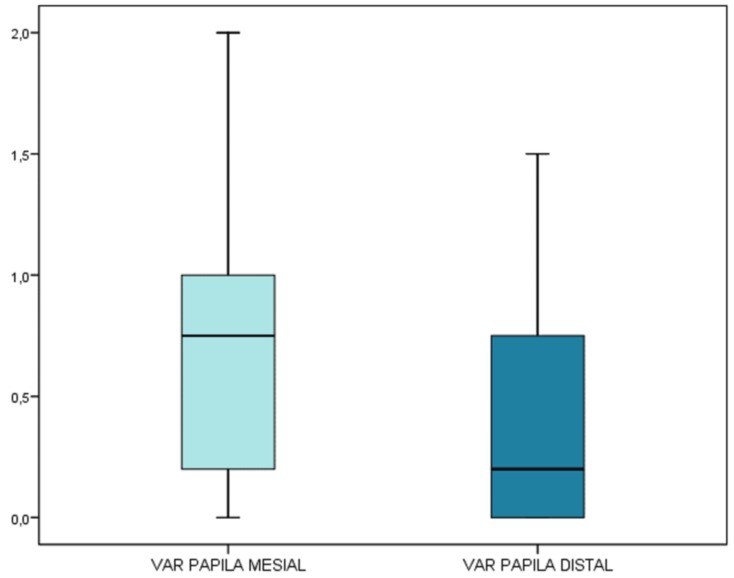
The gain at mesial level is significantly greater than at distal level.

The paired t-test indicated that the gain at mesial level was significantly greater than at distal level (*p*=0.017). A parallel nonparametric test (Wilcoxon) yielded the same result (*p*=0.022) (Fig. 4).

Differences in gain at papilla level were observed according to the structure adjacent to the implant (tooth, implant or pontic). In the total sample of 32 implants, the median mesial gain was 0.80, 0.30 and 0.40 mm when the structure adjacent to the implant was a tooth, implant or pontic, respectively (*p*=0.038; Kruskal-Wallis test). In the case of the unit crowns, the gain was significantly greater when the adjacent structure was a tooth (*p*=0.031; Mann-Whitney U-test). No statistically significant differences were noted in the case of the pontics (*p*=0.372; Mann-Whitney U-test).

At distal level, in the total 32 implants, smaller variations were recorded when there was no adjacent tooth or implant (median 0.05 mm). However, the similarity among the other three groups precluded statistical significance (*p*=0.311; Kruskal-Wallis test). The same results were obtained in relation to the unit crowns (*p*=0.400; Kruskal-Wallis test) and pontics (*p*=0.316; Kruskal-Wallis test).

In relation to gingival biotype, mesial papilla mean gains of 0.51 ± 0.48 mm and 0.87 ± 0.58 mm were observed for thin and thick biotypes respectively, and distal papilla gains of 0.24 ± 0.40 mm and 0.63 ± 0.51 mm for thin and thick biotypes respectively. Differences were only significant for distal papillaes.

Dimensional changes of the gingival margin

At buccal level, the mean increase in gingival margin was 0.56 ± 0.46 mm (range 0-1.50 mm) (95%CI 0.39-0.72), which likewise proved statistically significant (*p*<0.001; one-sample t-test). Although this parameter exhibited a near-normal distribution (p=0.068; Kolmogorov-Smirnov test), it was also described as a median: 0.50 mm (95%CI 0.2-0.8), with statistical significance again being observed (*p*<0.05). There were no significant differences in variation regarding implants forming or not forming part of pontics (*p*=0.593; Mann-Whitney U-test). Patient’s biotype did not significantly affect the variation of buccal margin, even though a bigger increment was seen in thick biotypes (thin biotype, mean: 0.41 ± 0.36 mm; normal-thick biotype, mean: 0.69 ± 0.51 mm) (*p*=0.114).

At lingual level, the mean increase was 0.33 ± 0.45 mm (range -0.50-1.60 mm) (95%CI 0.17-0.50), and therefore significant (*p*<0.001; one-sample t-test). The median increase in turn was 0.25 mm (95%CI 0-0.5), and proved nonsignificant. There were no significant differences in variation regarding implants forming or not forming part of pontics (*p*=0.293; Mann-Whitney U-test). No significant differences were found in lingual margin increase considering gingival biotype (thin biotype, mean: 0.24 ± 0.44 mm; thick biotype, mean: 0.42± 0,47 mm; *p*=0.551).

**Figure 5:**
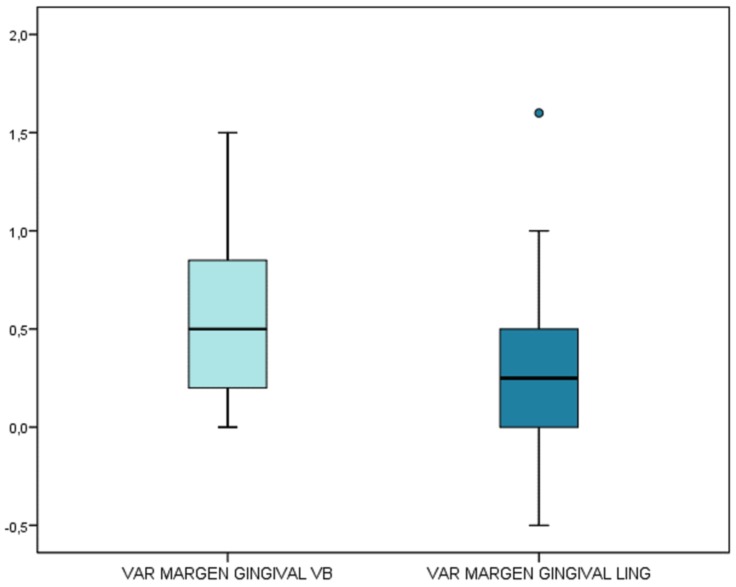
In the buccal zone the gain at marginal level is significantly more manifest.

On comparing the variation at buccal versus lingual level, both parametric and nonparametric testing confirmed the existence of significant differences (*p*=0.022; paired t-test, and *p*=0.024; Wilcoxon test). The increase in margin was significantly greater in the buccal zone (Fig. 5).

Changes in soft tissue volume 

Twenty-nine implants showed a mean increase in peri-coronal soft tissue volume of 64.7 mm3 (range 2.01-140.9 mm3). Three implants showed a decrease in peri-coronal soft tissue volume, with a mean decrease of -25.13 mm3, calculated as the mean of the values -46.1, 28.7 and -0.66 mm3.

Considering the total implants, the mean increase was 38.6 ± 47.3 mm3 and the median 17.48 mm3 (range -46.1-141.0), this reflecting the great dispersion of the variation values. The 95%CI of the median (4.02-60.32) indicated a statistically significant increase (*p*<0.05).

The implants with gingival volume loss corresponded to bridges of three crowns over two implants (2 cases). In these two cases, soft tissue volume loss was greater on including the pontic zone in the volumetric assessment and smaller on measuring peri-implant soft tissue volume without including the pontic zone.

The Mann-Whitney U-test identified no significant differences in volume change between unit crowns and crowns forming part of a bridge (*p*=0.654).

In both crowns and bridges, the zones exhibiting the greatest volume increments corresponded to the papillae of implants with adjacent teeth and the peri-coronal buccal gingival margin.

With regard to the increase in gingival volume at papilla level, the papillae of implants with adjacent teeth showed a greater volume increase than the papillae between implants. The papillae of distal implants with no adjacent tooth showed a minimum or no volume increase.

The gingival biotype of the patient strongly affected the overall soft tissue volume gain. Median volume increases of 5.2 mm3 and 58.2 mm3 were calculated for thin and thick biotypes respectively, being this difference statistically significant (*p*=0.024).

## Discussion

A number of studies have described different connective tissue fiber distributions around dental implants: parallel to the long axis of the implant (2,21), circular fibers forming a ring pattern (3,22), or inserted fibers (23,24). The connective tissue organizes around the abutment in the form of circular fibers (21), thereby stabilizing the tissue and contributing to reduce bone reabsorption (25).

The connective tissue is of crucial importance for stabilizing apical migration of the epithelium and affording protection against bone reabsorption. Discrepancies in diameter between the implant and abutment can establish a site where the circular connective tissue fibers may become retained. The use of convergent neck implants can result in more space for soft tissue stabilization. The circular fibers may be the key to soft tissue stability around the rehabilitation, preventing apical migration of the soft tissue and protecting the underlying bone (26).

A number of authors have proposed methods for preserving supporting bone through platform switching. The latter comprises a design in which the diameter of the abutment is smaller than that of the implant neck, and has been associated to lessened peri-implant bone loss compared with standard platform implants, thanks to the internally repositioned implant-abutment interface (mismatching), which limits peri-implant bone loss by keeping bacteria and infiltrating inflammatory cells away from the adjacent crestal bone (27).

In our study we found the increase in peri-implant soft tissues to be greater at the level of the papillae of the implants with an adjacent tooth (mesial or distal), and at the buccal margin. In contrast, the crowns of the pontics presented no gain or even showed tissue loss. The increased was seen to be less pronounced in the case of implants with no adjacent teeth or implants distally.

The median increase of the papilla at mesial level was 0.75 mm, versus 0.20 mm distal. The difference between the two levels was statistically significant. In the presence of an adjacent tooth, the papilla at mesial level showed a significantly greater increase. This effect was also observed in the unit crowns subgroup. The median gain at the buccal margin was 0.50 mm, versus 0.25 mm at lingual level – the difference again being statistically significant.

The mean volumetric change in the soft tissues measured between T0 and T1 was 38.62 mm3 (range -46.05-140.97; median 17.5 mm3) – the gain being statistically significant. In our study, the loss in soft tissue volume was associated to cases with bridges comprising three crowns over two implants. This was possibly related to the existence of a pontic.

Patient’s biotype influenced relevantly the changes occurring in the peri-implant soft tissues. In all the studied variables (papilla height, gingival margin and soft tissue volume) thin biotype yielded smaller changes. However, differences associated to biotype were only significant for total volume gain and distal papilla level.

The area measured was limited to the adjacent teeth in order to ensure great precision in the measurements obtained from the superimposed scans (28). By superimposing the scans we were actually only registering the external surface of the peri-implant zone under study. Therefore, and although visually (photographs and scans) the volumetric gains appeared to correspond to the peri-implant soft tissues at the peri-coronal margin, the fact that our study did not include tomographic acquisitions prevented us from fully discarding additional variations of the bone crest that may have influenced the volumetric findings of the scanner, which we attributed mainly to modifications of the peri-coronal soft tissues.

Despite our encouraging findings, further studies are needed, involving larger samples, different follow-up timepoints and controls in order to precisely analyze the volumetric changes around convergent collar implants with BOPT crowns, and their clinical relevance.

In conclusion, the use of crowns using the biologically oriented preparation technique (BOPT) over convergent collar implants resulted in a significant increase in peri-implant soft tissue volume 10 months after prosthetic loading.
